# Metal–Organic Framework Fluorescence Sensors for Rapid and Accurate Detection of Melamine in Milk Powder

**DOI:** 10.3390/bios13010094

**Published:** 2023-01-06

**Authors:** Mahmood Alizadeh Sani, Gholamreza Jahed-Khaniki, Ali Ehsani, Nabi Shariatifar, Mohammad Hadi Dehghani, Mohammad Hashemi, Hedayat Hosseini, Mohammad Abdollahi, Shokoufeh Hassani, Zahra Bayrami, David Julian McClements

**Affiliations:** 1 Division of Food Safety and Hygiene, Department of Environmental Health Engineering, School of Public Health, Tehran University of Medical Sciences, Tehran 1417614411, Iran; 2 Nutrition Research Center, Department of Food Science and Technology, Faculty of Nutrition and Food Sciences, Tabriz University of Medical Sciences, Tabriz 516615731, Iran; 3 Department of Environmental Health Engineering, School of Public Health, Tehran University of Medical Sciences, Tehran 1416634793, Iran; 4 Institute for Environmental Research, Center for Solid Waste Research, Tehran University of Medical Sciences, Tehran 1416634793, Iran; 5 Department of Nutrition, Faculty of Medicine, Mashhad University of Medical Sciences, Mashhad 9138813944, Iran; 6 Department of Food Science and Technology, National Nutrition and Food Technology Research Institute, Faculty of Nutrition Sciences and Food Technology, Shahid Beheshti University of Medical Sciences, Tehran 1985717443, Iran; 7 Department of Toxicology and Pharmacology, Faculty of Pharmacy, Tehran University of Medical Sciences, Tehran 1416634793, Iran; 8 Toxicology and Diseases Specialty Group, Pharmaceutical Sciences Research Center (PSRC), Tehran University of Medical Sciences, Tehran 1416634793, Iran; 9 Department of Food Science, University of Massachusetts, Amherst, MA 01003, USA

**Keywords:** metal–organic frameworks, coordinated polymers, fluorescence sensor, food adulterant, melamine

## Abstract

In this research, a simple, label-free, and ultra-sensitive fluorescent platform based on a metal–organic framework (MOF) has been developed to detect melamine in milk powder. This fluorescence sensor was fabricated from sensitized terbium (Tb)@NH_2_-MIL-253 (Al) MOF using a hydrothermal method that involved combining the green emission of Tb (λ_em_ = 545 nm) with the blue emission of NH_2_-MIL-253(Al) MOF (λ_em_ = 430 nm) under a single excitation wavelength (λ_ex_ = 335 nm). The fluorescence sensor was then used under optimized conditions (pH = 9.0; sensor concentration = 30 mg/L; response time = 30 s) to quantify melamine in milk powder. The accuracy, sensitivity, and reproducibility of this sensor were established compared to the high-performance liquid chromatography (HPLC) method. The linear range and lower limit of detection (LLOD, computed with 3σ/S) of the sensor were between 40–396.45 nM (equal to 25 µg/kg–0.25 mg/kg) and 40 nM (equal to 25 µg/kg), respectively, which is much less than the maximum residual level (MRL) for the detection of melamine in infant formula (1 mg/kg) and other foods/feeds (2.5 mg/kg). Additionally, the results had good agreement with the HPLC outcomes, suggesting that the NH_2_-MIL-253(Al) MOF sensing probe has great precision and repeatability. To conclude, the new fluorescence sensor developed in this study can accurately and sensitively detect melamine in food samples, which may be useful for screening for adulteration of milk powders and other foods.

## 1. Introduction

The safety of the global food supply chain is critical for ensuring the health and wellbeing of populations around the world. Foods may be intentionally or unintentionally contaminated with hazardous materials, such as bacterial pathogens, mycotoxins, pesticide residues, veterinary drugs, and heavy metals [1]. In some cases, adulterants are added to foods to fool the food industry or consumers into thinking a product is something that it is not, but these substances may harm human health [2].

Melamine is a nitrogenous heterocyclic organic chemical that has a variety of industrial applications, including in adhesives, plastics, flame retardants, cleaners, polymer resins, fertilizers, and catalysis [3,4]. In the food industry, melamine has been used illegally as an adulterant in dairy products, especially in milk powders and infant formula. The presence of this substance may lead to kidney stones and other health problems [5]. Accordingly, the World Health Organization (WHO) states that daily melamine intake should be less than 0.2 mg/kg body weight per day [6]. Melamine (MEL, 1,3,5-triazine-2,4,6-triamine) is used as an adulterant in dairy products because of its high nitrogen content (66.6%). The price of dairy products is often governed by their protein content, which is usually determined by measuring their nitrogen content, e.g., using Kjeldhal or Dumas methods. The addition of melamine to dairy products increases their total nitrogen content, which leads to an increase in the amount of protein detected using common assays [4]. Therefore, the detection, quantification, and monitoring of melamine in dairy and other food products, especially products related to infants, such as milk powder and infant formula, are necessary.

Food contaminants can be detected using many kinds of analytical methods, including high-performance liquid chromatography (HPLC), gas chromatography/mass spectrometry (GC/MS), ultra-performance liquid chromatography/mass spectrometry (UPLC/MS/MS), and various molecular-based methods [7]. These methods are typically effective, but there are some challenges to using them, such as high instrument costs, the need for trained and expert operators, the use of chemical solvents, complex sample preparation, and long preparation and analysis times, which limit their application [8]. In the last decade, sensors have been introduced to detect various food contaminants and hazardous materials. Sensor technologies are one of the most versatile methods available to detect and quantify contaminants. They can often be designed to overcome the challenges of conventional analytical methods. These techniques are often simple, cheap, user-friendly, rapid, sensitive, selective, and accurate [2]. Recently, nanosensors based on metal–organic frameworks (MOFs) have been developed for the detection and quantification of food contaminants, including mycotoxins, pesticides, drug residues, additives, and adulterants in foods. Moreover, progress has been made in improving the physical and chemical properties of MOF-based sensors, such as their robustness and functional performance [9,10].

Metal–organic frameworks (MOFs) are porous materials that consist of polymers coordinated to metal ions or metal clusters with multifunctional organic ligands [10,11]. Due to their polyhedral structure and controllable pore properties, they have been widely used as catalysts and for the design of sensing platforms [12,13]. The metals used to construct MOFs include iron, zirconium, aluminum, and lanthanides, which are suitable for the detection of different analytes. The detection of target molecules using MOFs requires a measurable signal, which are typically based on optical or electrochemical methods [14].

Nowadays, the use of nano/biosensors incorporated with MOFs, two-dimensional heterostructures, or bio/nanomaterials in the detection of various hazardous materials due to unique features, such as high accuracy, rapid response-time, user-friendly, stability in environmental conditions, simple operation, very low detection limits (micro, nano, pico, fento molar), and high sensitivity/selectivity has been considered [15]. In this regard, many studies have been focused on the synthesis and application of bio-based MOFs hybrids. Two strategies have been established for the fabrication of bio-based MOFs: (i) covalent binding based on different kinds of chemical reactions, and (ii) noncovalent connection mainly through hydrophobic and π-π stacking interactions [16]. Yu et al. (2021) developed a sensitive molecularly imprinted electrochemical aptasensor combined with gold nanoparticles (Au NPs) for the highly specific determination of melamine. The linear range between 10^−12^ M to 10^−4^ M with the detection limit of 6.7 × 10^−13^ M was obtained for detecting melamine [17]. In another study, Hu et al. (2017) developed and characterized a fluorescence probe for the detection of Ochratoxin A in corn samples based on an Fe_3_O_4_/g-C_3_N_4_/HKUST-1-MOFs aptasensor platform. The detection limit and linear range of this method were 2.57 ng/mL and 5–160 ng/mL, respectively [18].

**Scheme 1:**
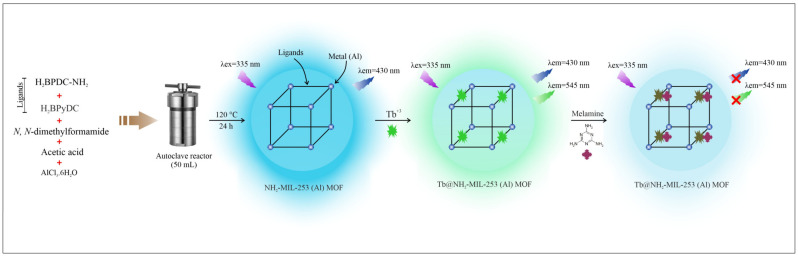
Schematic illustration for the synthesis of Tb@NH_2_-MIL-253 (Al) MOF fluorescence sensor and its interaction with melamine.

In the current study, we examined the potential of detecting and quantifying melamine in milk powder using Tb@NH_2_-MIL-253 (Al) MOF sensing probes as a dual emission fluorescence (FL) complex platform (Scheme 1) for the first time. We indicated that these probes are accurate, rapid, sensitive, user-friendly, simple, and convenient for melamine quantification over a wide concentration range with a linear response and low limit of detection. Finally, we showed that they could be used to detect melamine in real milk powder using a fluorescence spectrometer.

## 2. Experimental Section

### 2.1. Chemicals and Reagents

Aluminum chloride hexahydrate (AlCl_3_.6H_2_O), trichloroacetic acid (TCA), *N, N*-dimethylformamide (DMF), ethanol, methanol, acetonitrile, melamine, Tris-HCl buffer, and 2-amino-[biphenyl]-4,4′-dicarboxylic acid (H_2_BPDC-NH_2_) were purchased from Merck Co. (Germany). Terbium chloride hexahydrate (TbCl_3_.6H_2_O) was obtained from Sigma-Aldrich Co. LLC. (USA). 2,2′-[bipyridine]-5,5′-dicarboxylic acid (H_2_BPyDC) was obtained from Biosynth Carbosynth Co. (Staad, Switzerland). Milk powder sample was purchased from the pharmacy (Tehran, Iran). All the chemicals and reagents were obtained from commercial sources and utilized without any further purification.

### 2.2. Synthesis of NH_2_-MIL-253 (Al) MOF & Tb@NH_2_-MIL-253 (Al) MOF Sensing Probes

The NH_2_-MIL-253 (Al) MOF and Tb@NH_2_-MIL-253 (Al) MOF fluorescence platforms were synthesized using a hydrothermal method according to previous studies with some modifications [19,20]. Briefly, DMF (10 mL), acetic acid (860 μL, 15.0 mmoL), H_2_BPyDC (0.313 mmoL), and H_2_BPDC-NH_2_ (0.313 mmoL) were stirred for 15 min. Then, AlCl_3_.6H_2_O (0.625 mmoL) dissolved in DMF (5 mL) was incorporated into the above solution in an autoclave reactor (50 mL). The resulting mixture was then incubated for 24 h in an oven at 120 °C. After cooling at room temperature, the NH_2_-MIL-253 (Al) MOF white solid sample was collected by centrifugation (4000 rpm), Soxhlet extracted and washed with methanol, and then dried in a vacuum oven at 70 °C for 24 h.

The dual-emission Tb@NH_2_-MIL-253 (Al) MOF sensing platform was fabricated by mixing TbCl_3_.6H_2_O solution (50 mL, 2 mM in ethanol) and NH_2_-MIL-253 (Al) MOF (25 mg) and then keeping the samples for 24 h. The resulting precipitate was collected by centrifugation (4000 rpm), washed with ethanol to remove any unreacted species, and then completely dried in a vacuum oven at 70 °C for 24 h.

The synthesized NH_2_-MIL-253 (Al) MOF and Tb@NH_2_-MIL-253 (Al) MOF sensing platforms were characterized by field emission scanning electron microscopy (FE-SEM, MIRA III model, TESCAN Co, Czech Republic) at 10 kV to investigate the morphology of the fabricated MOFs; Fourier transform infrared spectroscopy (FTIR, AVATAR model, Thermo Co, USA) to investigate the molecular interaction of the sensing platform with spectral scan scope in the range of 4000–400 cm^−1^ and resolution of 4 cm^−1^; Brunauer–Emmett–Teller (BET, BELSORP MINI II model, BEL Co, Japan) to analyze the surface area under adsorptive N_2_; and X-ray diffraction (XRD, PW1730 model Philips Co, Netherlands,) to analyze the crystallinity degree of fabricated MOFs with Cu Kα radiation at 40 kV and 30 mA when 2θ = 5 to 80° to confirm the formation of NH_2_-MIL-253 (Al) MOF and Tb@NH_2_-MIL-253 (Al) MOF. Thermogravimetric analysis (TGA) was also carried out on an SDT Q600 analyzer (TA Instruments, USA) from 20 to 600 °C at a heating rate of 10 °C/min under air.

### 2.3. Fluorescence Response to Melamine

Before using Tb@NH_2_-MIL-253 (Al) MOF fluorescence probes to detect melamine, the experimental condition optimization including pH (3–12), incubation time (0–600 s), and nanosensor content (0–50 mg/L) was performed for the fluorescence probe using a fluorescence spectrometer (Perkin-Elmer LS-55, U.K).

Fluorescence probes (30 mg/L) were dispersed in deionized water and sonicated for 30 min. Aqueous solutions containing different concentrations (0–400 nM) of a stock standard solution of melamine (10 μg/mL in deionized water) were mixed with the fluorescence probes (Tb@NH_2_-MIL-253 (Al) MOF and NH_2_-MIL-253 (Al) MOF) and incubated at room temperature for 30 s. The final solutions were diluted to 3 mL using Tris-HCl buffer (pH = 9.0). After incubation for 30 s, the emission spectra were recorded from 350 to 600 nm at an excitation wavelength of 335 nm using a fluorescence spectrometer. The selectivity of the synthesized fluorescence platform towards melamine was investigated using other possible interferences (K^+^, Na^+^, Mg^2+^, Zn^2+^, Ca^2+^, Cl^−^, PO_4_^3−^, glucose, lactose, and ascorbic acid) and analogues (Cyanuric acid and Cyromazine) instead of melamine at the same preparation conditions. The intensity of their emission spectra was recorded with a fluorescence spectrometer.

### 2.4. Analysis of Melamine in Milk Powder Sample

The melamine level in a milk powder sample was measured using a standard addition method. At first, milk powder sample (1 g) was spiked with different concentrations of melamine standard solutions (0.5, 1, 2.5 and 5 mg/kg); the concentrations were selected based on the maximum residue level (MRL) of melamine in infant formula (1 mg/kg) and other foods/feeds (2.5 mg/kg) (World Health Organization, 2010). Then, 1% (*w*/*v*) trichloroacetic acid (2.5 mL) and acetonitrile (2.5 mL) were added to the spiked milk powder (1 g) and vortexed for 5 min to remove any proteins, lipids, and other organic compounds. Afterward, the spiked sample was sonicated for 20 min and centrifuged (9000 rpm for 5 min). The solution was then filtered through a 0.22 µm membrane filter, and the final solution was diluted to 5 mL with deionized water for further analysis [11,21]. Tb@NH_2_-MIL-253 (Al) MOF sensor results were compared with the results of the conventional method (HPLC). The HPLC analysis of melamine was carried out according to previous studies with some modification [22,23]. HPLC conditions included: Agilent 1200 series (Agilent technologies Inc., Santa Clara, CA, USA) on isocratic mod, UV detector at 240 nm, Zorbax XDB C_18_ column (250 × 4.6 mm, 5 µm), mobile phase consisting of water/methanol (70:30, *v*/*v*), flow rate of 1.0 mL/min, and 20 µL injection loop. Recovery was calculated according to the following formula:

Recovery (%) = ((Analyte concentration determined in spiked sample − Analyte concentration in unspiked sample)/Analyte concentration added to spiked sample) × 100(1)

### 2.5. Data Analysis

All experiments were carried out three times, and the mean and standard deviation were calculated.

## 3. Results and Discussion

### 3.1. Instrumental and Fluorescence Characteristics

**Figure 1:**
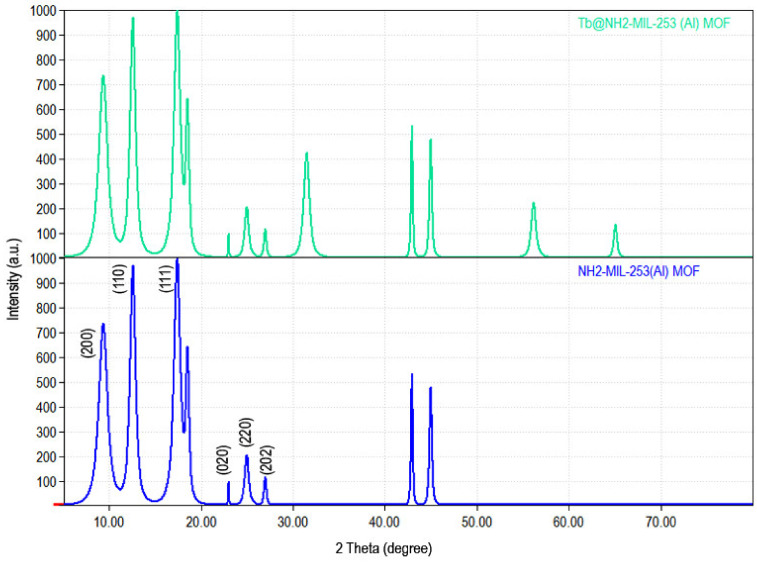
XRD patterns of Tb@NH_2_-MIL-253 (Al) MOF, and NH_2_-MIL-253 (Al) MOF fluorescence platforms.

The powder X-ray diffraction (PXRD) patterns were recorded to verify the crystallinity of NH_2_-MIL-253 (Al) MOF and Tb@NH_2_-MIL-253 (Al) MOF fluorescence probes (Figure 1). These products had fairly similar XRD patterns, and the crystal structure of NH_2_-MIL-253 (Al) MOF is not significantly changed after doping with Tb^3+^, suggesting the good dispersion of Tb^3+^ within the NH_2_-MIL-253 (Al) MOF matrix [20,24]. Moreover, no significant difference in the XRD patterns may be due to the low concentration of terbium [25]. The XRD pattern of the Tb@NH_2_-MIL-253 (Al) MOF (BET surface area 1261.27 m^2^ g^−1^) displayed the characteristic diffraction peaks of NH_2_-MIL-253 (Al) MOF, indicating that the framework of NH_2_-MIL-253 (Al) MOF was maintained without destruction after coordination of the Tb^3+^ ions by the bipyridine-chelating sites on the deprotonated ligands [26,27,28].

**Figure 2:**
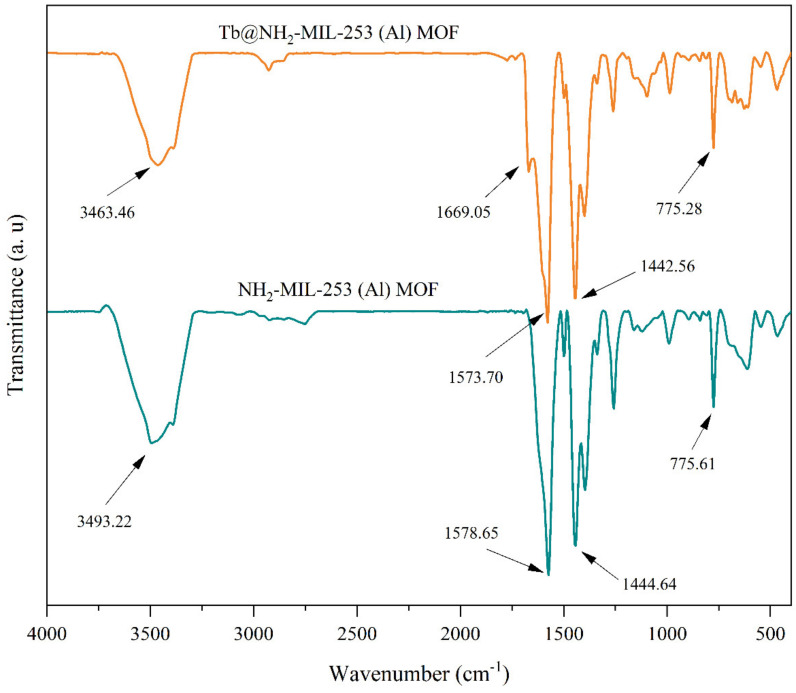
FTIR spectra of Tb@NH_2_-MIL-253 (Al) MOF, and NH_2_-MIL-253 (Al) MOF fluorescence platforms.

Similarly, the two products had similar FTIR spectra (Figure 2), which is also consistent with the good dispersion of Tb^3+^ in the NH_2_-MIL-253 (Al) MOF frameworks. The FT-IR spectra of Tb@NH_2_-MIL-253 (Al) MOF had an absorption peak at ~1669.05 cm^−1^ with a slight shift, which can be assigned to the stretching vibration of C=O (carbonyl), suggesting the successful insertion of Tb^3+^ ion into NH_2_-MIL-253 (Al) MOF network and the coordination of Tb^3+^ to the *N, N′* chelating sites in NH_2_-MIL-253 (Al) MOF [26,27,28].

**Figure 3:**
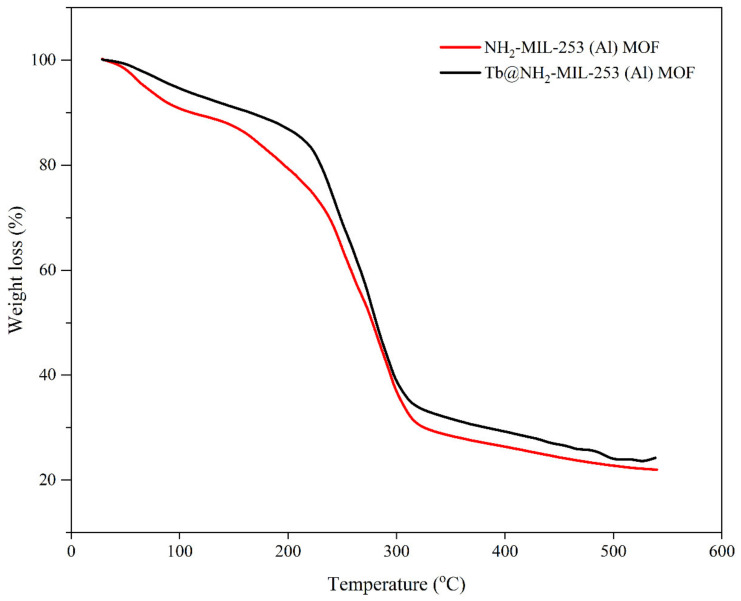
TGA profile of Tb@NH_2_-MIL-253 (Al) MOF, and NH_2_-MIL-253 (Al) MOF fluorescence platforms.

The TGA profiles, which provide information about the thermal decomposition of the MOFs, followed a fairly similar trend for the two samples (Figure 3). Typically, weight loss during heating before 300 °C is attributed to the evaporation of solvents and the decomposition of organic compounds, while decomposition of inorganic compounds occurs at higher temperatures.

**Figure 4:**
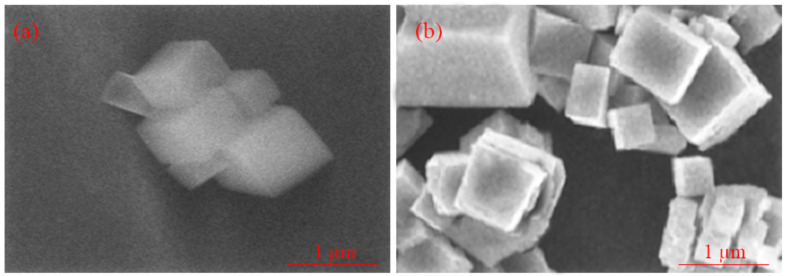
FE-SEM images of NH_2_-MIL-253 (Al) MOF (**a**), and Tb@NH_2_-MIL-253 (Al) MOF (**b**) fluorescence platforms.

The morphology of Tb@NH_2_-MIL-253 (Al) MOF and NH_2_-MIL-253 (Al) MOF was determined by FE-SEM and the images are shown in Figure 4a,b. The probes had a complex cubic-like morphology.

**Figure 5:**
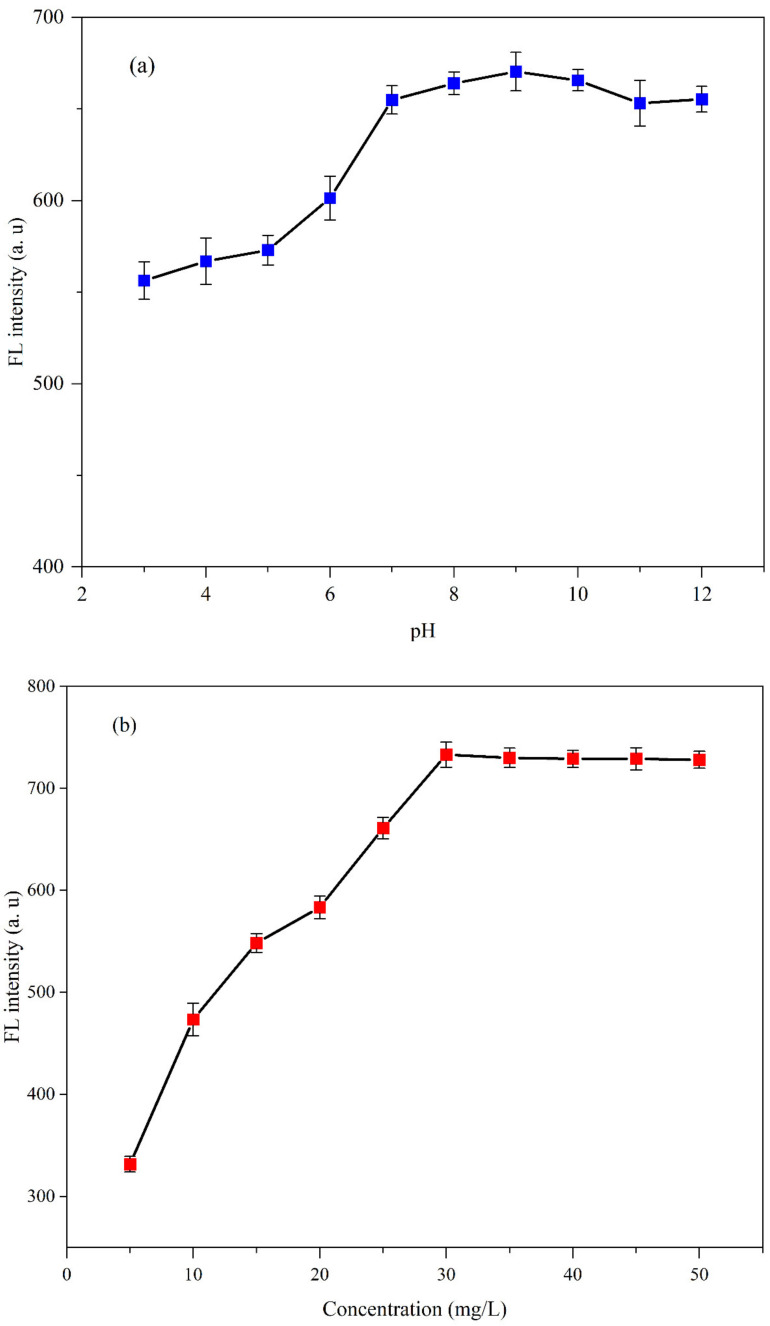

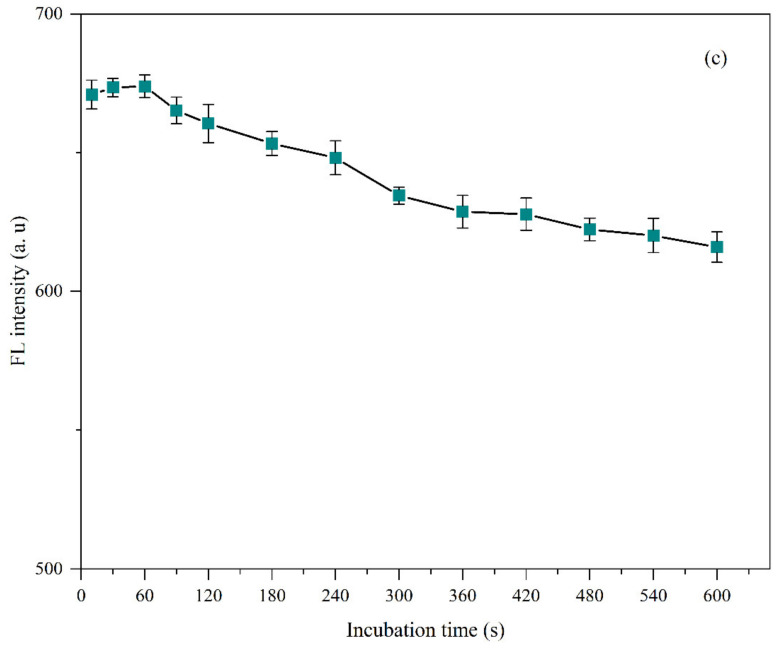
Effect of various pH (**a**), fluorescence platform content (**b**), and incubation time (**c**) of Tb@NH_2_-MIL-253 (Al) MOF sensing probe to detect melamine (experimental condition optimization).

The fluorescence excitation and emission spectra of NH_2_-MIL-253 (Al) MOF, and Tb@NH_2_-MIL-253 (Al) MOF in aqueous solution were analyzed under optimum sensing conditions (pH = 9.0, incubation time = 30 s and nanosensor content = 30 mg. L^−1^) in Tris-HCl buffer solution (10 mM, pH = 9.0) using a fluorescence spectrometer. As shown in Figure 5, the fluorescence intensity of the Tb@NH_2_-MIL-253 (Al) MOF platform was found to be dependent on the sensor concentration and pH values of the solution. The fluorescence probe showed the highest detection accuracy and fluorescence intensity at pH = 9 (Figure 5a), sensor concentration = 30 mg/L (Figure 5b), and incubation time = 30 s (Figure 5c). The response time of the fluorescent signal towards melamine was very fast, which can be completed within 1 min. However, with increasing sensor content from 0 to 50 mg/L in the medium, the fluorescence intensity remained almost unchanged or slightly decreased due to the phenomenon of saturation. On the other hand, the highest fluorescence intensity was observed in alkaline conditions. The pH value can impact the deprotonation or protonation process of Tb@NH_2_-MIL-253 (Al) MOF. The charge on the surface of the Tb@NH_2_-MIL-253 (Al) MOF would increase the deprotonation degree of the functional groups and may affect the electron–hole recombination process, triggering the fluorescence quenching [29].

**Figure 6:**
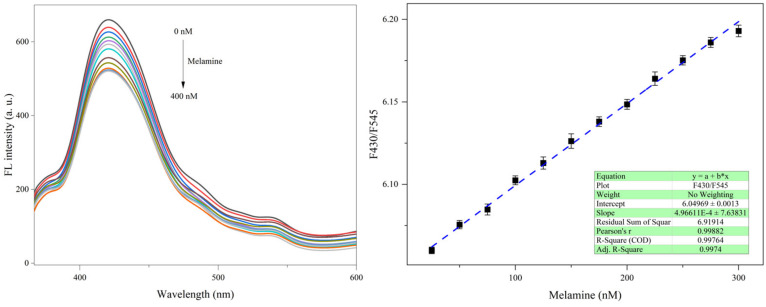
Fluorescence emission spectra of Tb@NH_2_-MIL-253 (Al) MOF sensing platform in the presence of different concentrations of melamine.

**Figure 7:**
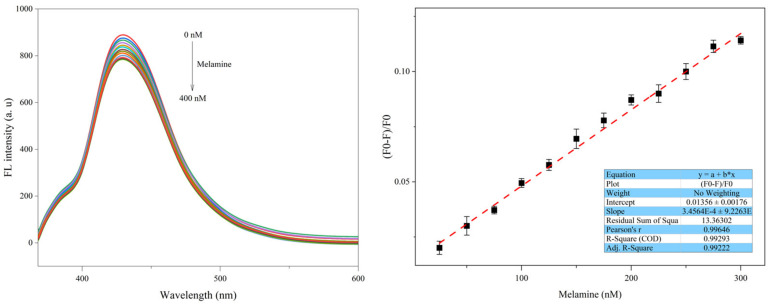
Fluorescence emission spectra of NH_2_-MIL-253 (Al) MOF sensing platform in the presence of different concentrations of melamine.

As indicated in Figure 6, Tb@NH_2_-MIL-253 (Al) MOF exhibited two characteristic emission peaks at 430 and 545 nm (^5^D_4_→^7^F_5_), which are related to NH_2_-MIL-253 (Al) MOF and Tb^3+^ ions, respectively, when excited at 335 nm [20,28]. In contrast, NH_2_-MIL-253 (Al) MOF only displayed a strong broad band centered around 430 nm at λ_ex_ = 335 nm (Figure 7), which can be assigned to the emission of H_2_BPDC-NH_2_ ligands [20,24].

**Figure 8:**
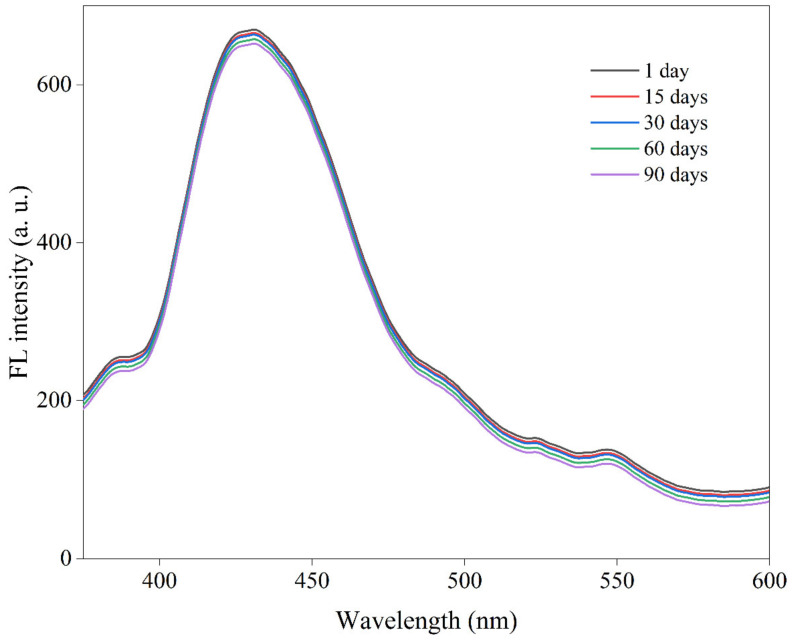
Fluorescence stability of Tb@NH_2_-MIL-253 (Al) MOF sensing platform for 90 days at room temperature.

The stability of fluorescence probe in aqueous dispersion was also investigated over 90 days at room temperature. As shown in Figure 8, the fluorescence intensity of the sensing probe was fairly stable throughout this storage period. This high fluorescence stability makes them especially suitable for sensing applications [24].

### 3.2. Luminescence Sensing of Melamine

In this series of experiments, the sensitivity and selectivity of the MOF-based fluorescence sensors were determined.

#### 3.2.1. Sensitivity of Sensors to Melamine

The fluorescent analysis of melamine was performed according to the optimized conditions based on Tb@NH_2_-MIL-253 (Al) MOF and NH_2_-MIL-253 (Al) MOF. As the results showed, the luminescence intensity of Tb@NH_2_-MIL-253 (Al) MOF at 430 and 545 nm gradually decreased with the increase in melamine content (0–400 nM). These changes were more prominent at 430 nm (NH_2_-MIL-253 (Al) MOF) than at 545 nm (Tb^3+^). When the melamine concentration was increased from 0 to 400 nM, the luminescence ratio (F_430_/F_545_) increased linearly. The quenching mechanism of quenchers such as melamine is typically investigated using the Stern–Volmer equation:

F_0_/F = 1 + *K*sv[C_q_] (2)

Here, F_0_ and F describe the fluorescence intensities of Tb@NH_2_-MIL-253 (Al) MOF without or with the quencher (melamine), *K*sv is the quenching constant [M^−1^], and [C_q_] is the concentration of the quencher (melamine). As a result, the melamine level could be quantitatively calculated using a standard curve method.

According to the LLOD = 3σ/S ratio, where S is the slope of the linear curve and σ is the standard deviation of the F_430_/F_545_ value of the blank sample, the lower limit of detection (LLOD) was determined to be 40 nM (25 µg/kg). This value is lower than the maximum residual level (MRL) for infant formula (1 mg/kg) and other foods/feeds (2.5 mg/kg). The linear range of the sensor was also obtained between 40–396.45 nM (25 µg/kg–0.25 mg/kg).

Compared to other studies conducted for measuring melamine (see Table 1), the Tb@NH_2_-MIL-253 (Al) MOF fluorescence sensor has great potential to detect melamine; as a result, the Tb@NH_2_-MIL-253 (Al) MOF sensor can be considered to be a sensitive means of quantifying melamine because it has a broad linear range and low detection limit.

#### 3.2.2. Selectivity of Sensors to Melamine

**Figure 9:**
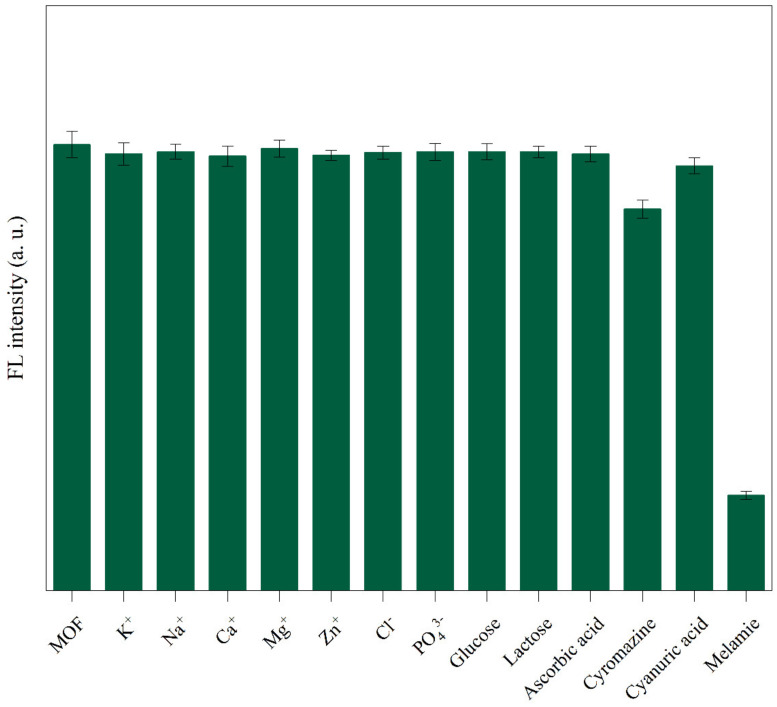
The relative fluorescence intensity of Tb@NH_2_-MIL-253 (Al) MOF aqueous suspension toward melamine or/and different interferences.

The selectivity of a sensing sensor towards specific target compounds and analogues is important because foods contain a diverse range of substances that could potentially interfere with the analysis. For this reason, we carried out an experiment to assess the selectivity of the sensors. As shown in Figure 9, some of the most common interferences (K^+^, Na^+^, Mg^2+^, Zn^2+^, Ca^2+^, Cl^−^, PO_4_^3−^, glucose, lactose, and ascorbic acid) and analogues (Cyanuric acid and Cyromazine) had a negligible effect on the fluorescence intensity of the designed Tb@NH_2_-MIL-253 (Al) MOF sensor, while melamine showed the highest effect on the fluorescence intensity of the Tb@NH_2_-MIL-253 (Al) MOF sensing probe. This revealed that Tb@NH_2_-MIL-253 (Al) MOF has a considerable selectivity and specificity towards melamine and appropriate application potential in real samples.

### 3.3. Sensing Mechanism

**Figure 10:**
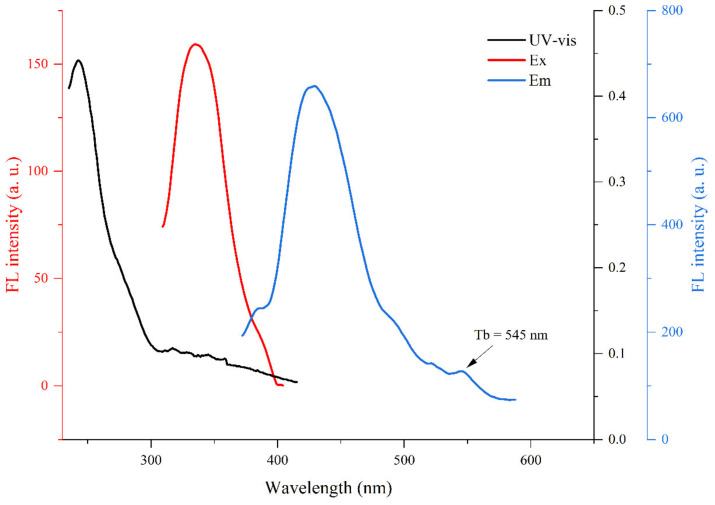
The excitation/emission spectra and UV-vis absorption of the Tb@NH_2_-MIL-253 (Al) MOF.

In this study, two fluorescence probes including Tb@NH_2_-MIL-253 (Al) MOF and NH_2_-MIL-253 (Al) MOF were used to detect melamine. As expected, the Tb@NH_2_-MIL-253 (Al) MOF exhibited a dual fluorescence emission spectrum with peaks around 430 nm (NH_2_-MIL-253 (Al) MOF) and 545 nm (Tb^3+^). In contrast, the spectra of the NH_2_-MIL-253 (Al) MOF only contained single peaks around ~430 nm. Typically, there are multiple mechanisms to describe the quenching of fluorescence signals, including the inner-filter effect (IFE), fluorescence resonance energy transfer (FRET), MOF framework collapse, and photoinduced electron transfer (PET) [20,38,39]. In the current study, the excitation and emission spectra of the Tb@NH_2_-MIL-253 (Al) MOF platform and the UV-visible absorption spectra of the Tb@NH_2_-MIL-253 (Al) MOF and melamine were compared (see Figure 10). An overlap between the spectra of the two components of the spectra was not observed. Consequently, the decrease in emission in the Tb@NH_2_-MIL-253 (Al) MOF sensing platform was not attributed to energy transfer or an inner filter effect [11,20]. Melamine can interact with the H_2_BPDC-NH_2_ ligand in the sensor via hydrogen bonding, donor–acceptor interactions, and π-stacking. As a result, the excitation energy absorbed by the ligand at λ_ex_ = 335 nm is suppressed by the melamine and disrupts the energy transfer of the ligand to the Tb^3+^ ion [11]. On the other hand, when the melamine binds to the Tb^3+^ ion owing to interactions between them, it causes the fluorescence intensity of the Tb@NH_2_-MIL-253 (Al) MOF sensing platform to be quenched [11,39,40]. The probable mechanism for terbium (Tb^3+^) quenching by melamine is the tendency of melamine to coordinate with metal ions through the multi-nitrogen heterocyclic ring [2,41]. In addition, activating organic ligands with functional groups (-NH_2_) leads to improving the water stability of MOFs and creates binding sites and electron transfer ability towards target molecules [24,42].

### 3.4. Detection of Melamine in Milk Powder Sample

To confirm the potential and accuracy of the fluorescence sensing system, the Tb@NH_2_-MIL-253 (Al) MOF platform was used to determine melamine in milk powder based on the standard addition method. Sample pretreatment was described in Section 2.4. Different concentrations of melamine standard solutions (0.5, 1, 2.5 and 5 mg/kg) were added to a milk powder sample, and then the fluorescence spectra were measured, and then the relevant data recorded (see Table 2). The Tb@NH_2_-MIL-253 (Al) MOF fluorescence sensor has great potential to detect melamine, because it has good accuracy, precision, response time, sensitivity, repeatability, and selectivity, and, on the other hand, the results from Tb@NH_2_-MIL-253 (Al) MOF fluorescence sensor were in good agreement with the results of the conventional method (HPLC). The results indicated that melamine was not detected in the unspiked milk powder by the Tb@NH_2_-MIL-253 (Al) MOF sensing platform. The results of luminescence detection exhibited that the relative standard deviation (RSD%, n = 3) of spiked milk powder sample with melamine was between 1.8% and 3.3% and the recovery percentage was obtained between 98.5% and 102.6%, indicating the high precision of this method, which was reliable with the results from HPLC, suggesting that the Tb@NH_2_-MIL-253 (Al) MOF florescence platform has good accuracy, precision, and reproducibility. Accordingly, the Tb@NH_2_-MIL-253 (Al) MOF sensing platform could have great feasible and potential applicability for the quantitative determination of melamine in real milk powder samples.

## 4. Conclusions

Porous NH_2_-MIL-253 (Al) MOF was synthesized using a one-pot reaction hydrothermal method and then used to encapsulate Tb^3+^ ions to fabricate the Tb@NH_2_-MIL-253 (Al) MOF sensing platform. The resulting Tb@NH_2_-MIL-253 (Al) MOF fluorescence platform had dual emission signals, which enabled the fluorescence detection of melamine in a real milk powder sample. The Tb@NH_2_-MIL-253 (Al) MOF sensing platform had good sensitivity and selectivity towards melamine. The florescence intensity was decreased at 430 and 545 nm, upon the addition of melamine. The Tb@NH_2_-MIL-253 (Al) MOF sensing system displayed a wide linear range between 40–396.45 nM, a lower limit of detection (40 nM), and a quick response time (30 s). More importantly, the Tb@NH_2_-MIL-253 (Al) MOF could be utilized for the detection of melamine in real milk powder samples with good recovery (98.5–102.6%) and RSD (1.8% to 3.3%) values, which had a good agreement with the results from HPLC. Accordingly, the Tb@NH_2_-MIL-253 (Al) MOF sensing platform can be considered as having potential applications for detecting melamine in milk powders. However, further research is required to establish its efficacy for application to other foods, as well as to ensure that it can be converted into a practical sensing technology for widespread application in the food industry.

## Figures and Tables

**Table 1:** The comparison of reported sensing systems for the detection of melamine.

Methods	Sensing Materials	LR	LOD	Ref.
Fluorescence	Tb@NH_2_-MIL-253 (Al) MOFs	40–396.45 nM	40 nM	This study
Colorimetric	Ag NPs	0.2–2 ppm	0.07 ppm	[30]
Fluorescence	Carbon nanodots-Hg^2+^	1–20 μM	0.3 μM	[31]
Fluorescence	UiO-66-NH_2_@Ru MOFs	0.27–110 μM	90 nM	[11]
Fluorescence	Ag NPs/Carbon dots	0.05–0.35 ppm	0.017 ppm	[32]
Fluorescence	Cadmium selenide (CdSe) quantum dots (QDs)	0.01 nM to 60 μM	0.013 nM	[33]
Fluorescence/aptasensor	Sensitized terbium (III)	1–10 ppm	0.02 ppm	[34]
Fluorescence	Acrylate citric acid	3.96–70.73 nM	0.23 nM	[35]
HPLC	-	0.015–0.1261 μg/mL	0.015 μg/mL	[36]
HPLC	-	0.017–0.052 μg/mL	0.017 μg/mL	[37]

LOD: Limit of detection, LR: Linear range, Tb: Terbium, Ag NPs: Silver nanoparticles, MOFs: Metal organic frameworks, HPLC: High-performance liquid chromatography, ppm: Parts per million.

**Table 2:** Application of the proposed Tb@NH2-MIL-253 (Al) MOF sensing platform and HPLC for the detection of melamine in milk powder sample.

Sample	Spiked Melamine (mg/kg)	Founded Melamine by Tb@NH_2_-MIL-253 (Al) MOF (mg/kg)	Recovery (%)	RSD (%, n = 3)	Founded Melamine by HPLC (mg/kg)	Recovery (%)	RSD (%, n = 3)
Milk powder	0	-	-	-	-	-	-
	0.5	0.480 ± 0.05	99.5 ± 2.66	2.2	0.47 ± 0.1	94 ± 3.33	1.5
	1	1.026 ± 0.08	102.6 ± 1.33	1.9	1.05 ± 0.05	105 ± 2.66	2.8
	2.5	2.462 ± 0.11	98.5 ± 2.30	3.3	2.505 ± 0.3	100 ± 2.50	3.1
	5	5.004 ± 0.33	100.2 ± 0.97	1.8	5.15 ± 0.25	103 ± 1.20	2.0

Tb: Terbium, MOFs: Metal organic frameworks, HPLC: High-performance liquid chromatography, RSD: Relative standard deviation.

## Data Availability

Not applicable.
